# Commentary: Integrative Modeling and the Role of Neural Constraints

**DOI:** 10.3389/fpsyg.2017.01531

**Published:** 2017-09-05

**Authors:** Brice Bantegnie

**Affiliations:** Department of Analytic Philosophy, Institute of Philosophy (ASCR) Prague, Czechia

**Keywords:** mechanistic explanation, functional analysis, mechanistic integration, reverse inference, neural plasticity, neural networks

Daniel Weiskopf has recently argued that the mechanistic integration of cognitive and mechanistic models can't help resolve the potential conflicts between these two types of models (Weiskopf, 2016). I will show that his argument rests on a mistaken picture of mechanistic integration.

In cognitive science, the “Many-Models Problem” (Weiskopf, 2016) arises when one believes in three incompatible propositions: that a cognitive and a neurocognitive model are both true of a target system, that they are made true by the way the world is and that they ascribe incompatible properties to this system. According to the received view, cognitive explanations are functional (Cummins, 1985). To explain our having a given capacity, one puts forward a model representing a system constituted of distinct mechanisms—*functionally* characterized, i.e., by the role they play in the system—causally related to one another. For example, Donald Broadbent explained the selectivity of attention by hypothesizing that a *filter* operated a selection on the input in order to reduce the quantity of information transmitted upstream so that it could be transmitted through a limited capacity channel (Broadbent, 1958). A cognitive model is accurate to the extent that it predicts the data produced by subjects in experimental contexts. There is therefore no guarantee that the structure ascribed to the target system will match the one ascribed by a neurocognitive model. Sometimes, a Many-Model problem may arise.

How can mechanistic integration help? Mechanisms are sets of entities and their activities organized in such a way that they produce, underlie, or maintain a phenomenon (Craver and Darden, 2013). To explain a phenomenon mechanistically one puts forward a model representing a system constituted of distinct mechanisms structurally characterized (Craver, 2007). For example, to explain the rat's capacity for spatial memory, researchers have put forward a hierarchical model bottoming out in the phenomenon of Long Term Potentiation (a long-lasting strengthening of a synapse) of the neurons of the dentate gyrus of the rat's hippocampus, and a mechanism underlying this phenomenon in which Ca^2+^ ions in the post-synaptic neuron play a key role (Granger and Nicoll, 2014). A cognitive and a neurocognitive model are mechanistically integrated when the mechanisms functionally characterized by the former are identical to the mechanisms structurally characterized by the later and when the former is a sketch of the later (Piccinini and Craver, 2011). In this situation, the two models do not ascribe incompatible properties to the same system.

Weiskopf thinks that in many cases such an integration is not forthcoming. In this situation, applying a neural plausibility constraint to cognitive models is misguided. A mechanistic integration has two steps: the development of the cognitive and the neurocognitive model; the mapping of the functional characterizations to the structural characterizations (the “localization” step). Here is in a nutshell Weiskopf's argument. First, “if localization is successful, cognitive functions will end up being assigned to distinct spatially and structurally well-defined components of the brain,” that is, a one-to-one mapping is a necessary condition for localization. Second, recent evidence from systems neuroscience in which brain functions—i.e., cognitive functions—are to be understood in terms of whole networks, shows that some brain regions are multifunctional. To start with, anatomically disjoint brain regions can contribute “processing to partially anatomically disjoint structural networks” and “support different modes of processing within one and the same anatomical network.” Moreover, “extrinsic modulations of regional functions [are] commonplace.” Third, it follows that localization will fail in many cases. Consequently, in many cases, mechanistic integration will fail. I won't discuss Weiskopf's neuroscientific evidence (Anderson, 2010; Sporns, 2010). Instead, I will argue that mechanists are not, *by being mechanists*, committed to this view of localization. Weiskopf doesn't provide any textual evidence that mechanists subscribe to it but, in any case, this would be an additional commitment on their part.

I will first note that Weiskopf's second step wrongly assumes that mechanists have to say that the brain regions to be mapped are anatomical brain regions (“dentate gyrus of the hippocampus,” “inferotemporal cortex”). Mechanists traffic in entities, their activities, and the organization thereof: there is no reason why the entities of the higher-level couldn't be those of systems neuroscience (networks) instead of those of anatomy. This reply won't do, however, as Weiskopf could reformulate his argument in terms of networks: the evidence he provides does show that networks are multifunctional. Mechanists, however, are not committed to the view of localization Weiskopf starts with. It is open to them to say that the same entity (here, a network) performs distinct functions, as long as they explain these functions by invoking the distinct activities of this entity's parts and their organization. There being extrinsic modulations of regional functions doesn't add anything to the argument. This amounts to saying that for the mapping to be possible, a *modulator* will have to be part of the cognitive model. But there might be good psychological evidence for this.

Weiskopf gives two arguments in favor of a one-to-one mapping being a necessary condition for localization. First, if two or more brain regions performing distinct functions overlap, these functions are not always independently modifiable. This casts doubt on the reality of distinct cognitive mechanisms performing these distinct functions. However, Weiskopf assumes once more that the brain regions to be mapped are anatomical brain regions. We would need to be shown evidence that *networks* overlap. Second, reverse inference—the inference from brain activity to psychological states—should be possible. But, according to the mechanistic view, were future brain imaging techniques to give us a window on the activities of parts of the brain and their interactions then neuroscientists would have the data on which to base reverse inferences. And this may very well happen.

Weiskopf calls for humility in applying constraints such as neural plausibility. I don't know whether mechanists lack humility. But, their work should, hopefully, help give cognitive neuroscience its proper place inside the cognitive sciences.

## Author contributions

The author confirms being the sole contributor of this work and approved it for publication.

### Conflict of interest statement

The author declares that the research was conducted in the absence of any commercial or financial relationships that could be construed as a potential conflict of interest.

## References

[B1] Anderson M. L. (2010). Neural reuse: a fundamental organizational principle of the brain. Behav. Brain Sci. 33:245 10.1017/S0140525X10000853 20964882

[B2] Broadbent D. E. (1958). Perception and Communication. London, UK: Pergamon Press.

[B3] Craver C. F. (2007). Explaining the Brain: Mechanisms and the Mosaic Unity of Neuroscience. Oxford, UK: Oxford University Press; Clarendon Press.

[B4] Craver C. F. Darden L. (2013). In Search of Mechanisms: Discoveries across the Life Sciences. Chicago, IL: University of Chicago Press.

[B5] Cummins R. (1985). The Nature of Psychological Explanation. Cambridge, MA: MIT Press.

[B6] Granger A. J. Nicoll R. A. (2014). Expression mechanisms underlying long-term potentiation: a postsynaptic view, 10 years on. Philos. Trans. R. Soc. B Biol. Sci. 369:20130136. 10.1098/rstb.2013.0136 24298139 PMC3843869

[B7] Piccinini G. Craver C. (2011). Integrating psychology and neuroscience: functional analyses as mechanism sketches. Synthese 183, 283–311. 10.1007/s11229-011-9898-4

[B8] Sporns O. (2010). Networks of the Brain. Cambridge, MA: MIT Press.

[B9] Weiskopf D. A. (2016). Integrative modeling and the role of neural constraints. Philos. Sci. 83, 674–685. 10.1086/687854

